# Regulation of barrier immunity and homeostasis by integrin‐mediated transforming growth factor *β* activation

**DOI:** 10.1111/imm.13162

**Published:** 2019-12-25

**Authors:** Craig P. McEntee, Sezin Gunaltay, Mark A. Travis

**Affiliations:** ^1^ Lydia Becker Institute for Immunology and Inflammation Manchester UK; ^2^ Wellcome Trust Centre for Cell‐Matrix Research Manchester UK; ^3^ Faculty of Biology, Medicine and Health Manchester Collaborative Centre for Inflammation Research (MCCIR) Manchester Academic Health Sciences Centre University of Manchester Manchester UK

**Keywords:** immune system, integrins, intestine, lung, skin, transforming growth factor‐*β*

## Abstract

Transforming growth factor *β* (TGF‐*β*) is a multifunctional cytokine that regulates cell growth, differentiation, adhesion, migration and death dependent on cell type, developmental stage, or tissue conditions. Various cell types secrete TGF‐*β*, but always as an inactive complex. Hence, for TGF‐*β* to function, this latent complex must somehow be activated. Work in recent years has highlighted a critical role for members of the *α*_v_ integrin family, including *α*_v_ *β*_1_, *α*_v_ *β*_3_, *α*_v_ *β*_5_, *α*_v_ *β*_6_ and *α*_v_ *β*_8_ that are involved in TGF‐*β* activation in various contexts, particularly at barrier sites such as the gut, lung and skin. The integrins facilitating this context‐ and location‐specific regulation can be dysregulated in certain diseases, so are potential therapeutic targets in a number of disorders. In this review, we discuss the role of TGF‐*β* at these barrier sites with a focus on how integrin‐mediated TGF‐*β* activation regulates tissue and immune homeostasis, and how this is altered in disease.

## Graphical abstract

TGF‐*β* is a crucial cytokine in regulation of the immune system, especially at barrier sites. Many cell types can produce TGF‐*β*, but always as an inactive complex that requires activating to be functional. This review highlights vital pathways that activate TGF‐*β* in the intestine, lung and skin, with a focus on how integrins control TGF‐*β* activity in a context‐specific manner.

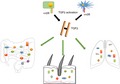

**Abbreviations**
COPD: chronic obstructive pulmonary disease
DCs: dendritic cells
IBD: inflammatory bowel disease
Ig: immunoglobulin
IPF: idiopathic pulmonary fibrosis
IRF: interferon regulatory factor
KLRG1^+^: killer-cell lectin like receptor G1
LAP: latency‐associated peptide
LLC: large latent complex
LTBP: latent TGF‐*β* binding protein
RALDH: retinal dehydrogenase
SLC: small latent complex
Smad: Similar to mothers against decapentaplegic
TGF‐*β*: transforming growth factor‐*β*
Th cell: T helper cell
Treg cells: regulatory T cells
Trm cells: tissue‐resident memory cells

## Introduction

**Figure 1:**
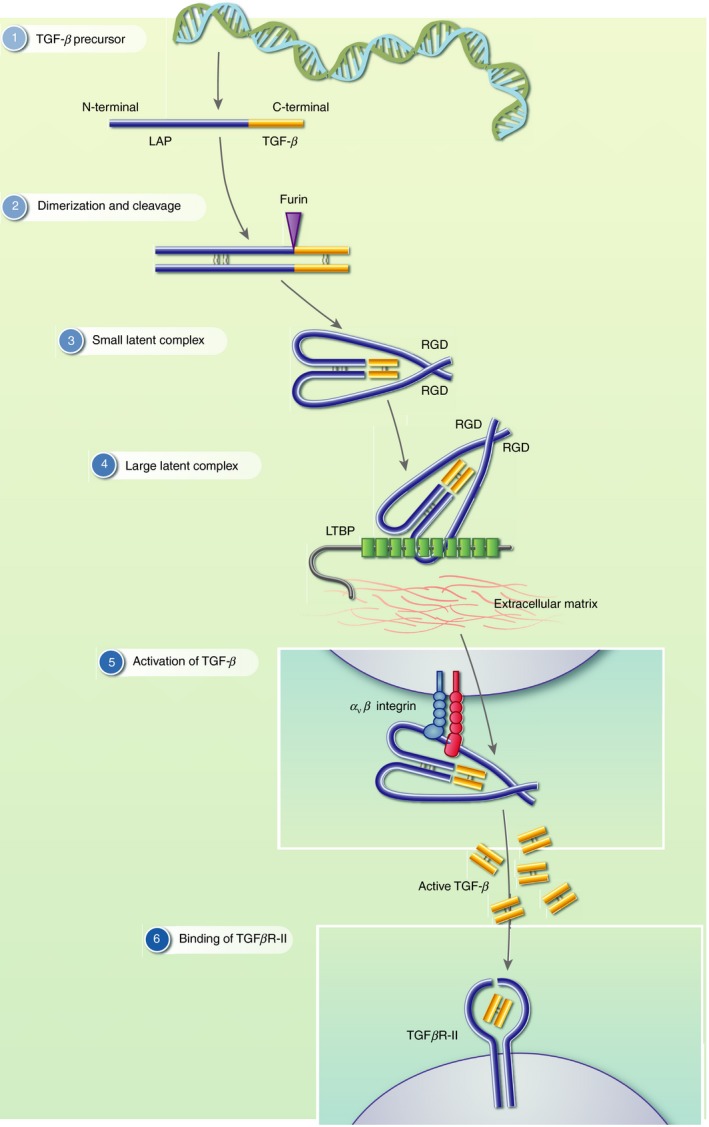
Structure of latent transforming growth factor‐*β* (TGF‐*β*) and activation by integrins. (1) TGF‐*β* is synthesized as a precursor that comprises an N‐terminal latency‐associated peptide (LAP) and a C‐terminal active TGF‐*β* moiety. (2) LAP–TGF‐*β* forms a homodimeric propeptide complex, which is cleaved by the protease furin intracellularly. (3) The small latent complex (SLC) comprises the cleaved LAP non‐covalently bound to active TGF‐*β* upon secretion. (4) Often, the SLC covalently associates with latent TGF‐*β* binding protein (LTBP) to form the large latent complex (LLC) together with the extracellular matrix. (5) *α*_v_ integrins important activators of TGF‐*β* binds to LAP at an arginine‐glycine‐aspartic acid (RGD) site, leading to the dissociation of LAP and the release of active TGF‐*β*. (6) Active TGF‐*β* first binds to the TGF*β*RII dimer.

Transforming growth factor‐*β* (TGF‐*β*) is part of a large protein family comprising 33 members, including three isoforms of TGF‐*β* (TGF‐*β*_1_, ‐*β*_2_ and ‐*β*_3_) as well as bone morphogenetic proteins, growth and differentiation factors such as Nodal, activins/inhibins, Müllerian inhibiting substance/anti‐Müllerian hormone and Lefty.^1^, ^2^ The binding of TGF‐*β* to TGF‐*β* receptors initiates a cascade of intracellular signals, which can proceed along both Smad [homologues of the Sma and mothers against decapentaplegic (Mad) proteins found in *Caenorhabditis elegans* and *Drosophila*, respectively] ‐dependent and ‐independent pathways as reviewed elsewhere.^3^, ^4^ Responses triggered by TGF‐*β* signalling can regulate cell growth, differentiation, adhesion, migration and death depending on target cell type, developmental stage or tissue environment.^1^, ^2^, ^5^ Due to this pleiotropy, the functionality of TGF‐*β* is tightly regulated and all three isoforms are synthesized as inactive precursors comprising an N‐terminal latency‐associated peptide (LAP) and a C‐terminal active TGF‐*β* moiety (Fig. 1). LAP–TGF‐*β* forms a homodimeric pro‐peptide complex, which is cleaved by the protease furin intracellularly.^6^, ^7^ However, following this initial cleavage event, LAP remains non‐covalently associated with active TGF‐*β*, which is secreted as a small latent complex (SLC). The configuration of this SLC is such that LAP masks the active TGF‐*β* fragment, thereby blocking its receptor binding sites and rendering it inactive (Fig. 1). Often, the SLC covalently associates with latent TGF‐*β*‐binding protein (LTBP) to form the large latent complex (LLC). Upon secretion, LTBP interacts with other components of the extracellular matrix and can be covalently cross‐linked to some matrix proteins.^8^, ^9^, ^10^, ^11^ However, in order for TGF‐*β* to bind to its receptors and trigger signalling, it must first be separated from LAP. Various mechanisms by which this process could occur have been proposed, including extremes of heat, acidic pH and reactive oxygen species, as well as the activities of serine proteases, matrix metalloproteases and thrombospondin‐1. Furthermore, recent compelling evidence suggests that key activators of TGF‐*β* are integrins (Fig. 1).^12^ These molecules are part of a large family of heterodimeric transmembrane receptors and consist of an *α* and a *β* subunit.^13^ Members of the α_v_ integrin family, including *α*_v_ *β*_1_, *α*_v_ *β*_3_, *α*_v_ *β*_5_, *α*_v_ *β*_6_ can bind to the tri‐amino integrin‐binding motif RGD in LAP, while simultaneously interacting with cytoskeletal proteins to facilitate mechanical fragmentation of LAP.^14^, ^15^, ^16^, ^17^, ^18^, ^19^ Although integrin *α*_v_ *β*_8_ also binds to LAP via RGD, the activation occurs via non‐cytoskeletal‐dependent mechanisms.^20^ While the integrin‐binding RGD motif is present in the latent form of TGF‐*β*_1_ and TGF‐*β*_3_, it is not present in TGF‐*β*_2_.^12^ Several of these TGF‐*β*‐activating integrins play fundamental roles in the preservation of normal tissue functions at barrier sites including the gut, lung and skin as discussed below.

In this brief review, we discuss critical roles of integrin‐mediated activation of latent TGF‐*β* at three distinct barrier sites – the gut, lung and skin – highlighting the importance of this process in both healthy and disease states and discussing the therapeutic potential for these pathways.

## Activation and function of TGF‐*β* in the gastrointestinal tract

Maintenance of immune equilibrium in the gastrointestinal tract is complex and multi‐faceted as harmful antigens originating from enteric pathogens must be distinguished from those that are innocuous and derived from diet or the microbiota. Central to this balance between effector and regulatory responses is TGF‐*β*, derived from a multitude of cells including regulatory T (Treg) cells, dendritic cells (DCs) and intestinal epithelial cells. Indeed, TGF‐*β* plays a central role in shaping the immunological landscape of the gut as it is an essential factor involved in the differentiation of both Treg and T helper type 17 (Th17) cells.^12^ Furthermore, the ability of effector T cells to respond to TGF‐*β* is also important in their suppression by Treg cells in models of intestinal inflammation.^21^ In addition to its effects on T cells, TGF‐*β* can also induce immunoglobulin A (IgA) production by intestinal plasma cells, which in turn helps to shape the composition of the microbiota to one which favours a tolerogenic environment.^22^, ^23^, ^24^, ^25^ Given this functional diversity, it is important to understand the mechanism(s) by which TGF‐*β* activation occurs in the intestine and how such processes regulate the different responses induced by TGF‐*β*.

### TGF‐*β* activation by tolerogenic intestinal DCs

Dendritic cells expressing the integrin CD103 (integrin *α*_E_) appear to be particularly important in promoting intestinal tolerance owing to their co‐expression of Vitamin A‐metabolizing retinal dehydrogenase (RALDH) enzymes and TGF‐*β*, which enables them to promote the differentiation of Treg cells.^26^, ^27^ Furthermore, these CD103^+^ DCs are capable of activating latent TGF‐*β* via their expression of the integrin *α*_v_ *β*_8_, a feature which is essential for the maintenance of normal intestinal immune function.^28^, ^29^, ^30^, ^31^, ^32^ Additionally, *α*_v_ *β*_8_‐mediated TGF‐*β* activation by DCs can modulate different intestinal CD4^+^ T helper cell responses. For instance, integrin *α*_v_ *β*_8_‐mediated TGF‐*β* activation by DCs inhibits the differentiation of Th2 cells during infection with the intestinal parasite *Trichuris muris*, with mice lacking this pathway showing exacerbated Th2 cell numbers and protection from chronic infection.^33^ However, the ability of DC‐mediated TGF‐*β* activation to control responses to intestinal parasites is dependent on the pathogen, as recent data have shown that deletion of *α*_v_ *β*_8_ on DCs results in delayed expulsion of *Trichinella spiralis*. This delayed expulsion appears to be the result of decreased TGF‐*β*‐dependent Th17 cell differentiation, which resulted in impaired intestinal contractility – an important mechanism by which *T. spiralis* is expelled.^34^ Additionally, reduction in Th17 cell numbers in the gut and lymphoid tissues of mice lacking integrin *α*_v_ *β*_8_ expression on DCs has been shown to correlate with complete protection from experimental autoimmune encephalomyelitis, a Th17‐mediated pathology with a similar immune and pathological profile to multiple sclerosis.^35^, ^36^, ^37^ Taken together, these studies show a key role for integrin *α*_v_ *β*_8_‐mediated TGF‐*β* activation by DCs in controlling CD4^+^ T‐cell responses in the gut. Additionally, the expression of integrin *α*_v_ *β*_8_ by Peyer's patch‐derived DCs, and their resulting ability to activate latent TGF‐*β*, was shown by Reboldi *et al.* 25 to be essential for IgA class switching of activated B cells within these intestinal lymphoid structures, showing a broader role for DC expression of integrin *α*_v_ *β*_8_ in regulating adaptive immunity in the gut.

Integrin *α*_v_ *β*_8_ is also expressed by certain human intestinal DC subsets. However, whereas integrin *α*_v_ *β*_8_ is preferentially expressed by interferon regulatory factor (IRF) 8‐dependent CD103^+^ CD11b^+^ intestinal DCs in mice,^32^, ^38^ expression of *α*_v_ *β*_8_ is restricted to IRF4‐dependent CD1c^+^ and not IRF8‐dependent CD141^+^ DCs in humans and is significantly enhanced during active inflammation and in response to Toll‐like receptor ligands.^39^, ^40^

Perturbations in TGF‐*β* activation pathways are also observed in inflammatory bowel disease (IBD). While total concentrations of latent TGF‐*β* are elevated during IBD,^41^ decreased expression of the TGF‐*β*‐activating integrin *α*_v_ *β*_8_ on mature tissue macrophages and infiltrating pro‐inflammatory monocytes is a feature of intestinal inflammation in humans.^42^ Interestingly, this is in contrast to the pattern of expression observed on DCs isolated from intestinal biopsies from IBD patients, which exhibit enhanced levels of *α*_v_ *β*_8_ compared with those from healthy subjects.^40^ Although the mechanism(s) underlying the dichotomous expression of this TGF‐*β*‐activating integrin during intestinal inflammation remains to be elucidated, work to date suggests that alterations in the capacity of specific cell types to activate latent TGF‐*β* is a feature of IBD.

### Suppression of intestinal inflammation by Treg cells

A reduction in the number of peripheral Treg cells, or alterations in their functionality, have also been reported in IBD.^43^, ^44^ Recent work has shown that Treg cells also activate TGF‐*β* through expression of integrin *α*_v_ *β*_8_.^45^ However, unlike DCs, conditional deletion of *α*_v_ *β*_8_ on Treg cells did not perturb immune quiescence under steady‐state conditions. Instead, ablation of integrin *α*_v_ *β*_8_ expression on Treg cells prevents these cells from resolving established, ongoing intestinal inflammation, with activated killer‐cell lectin like receptor G1 (KLRG1^+^) effector Treg cells expressing high levels of integrin *α*_v_ *β*_8_ rapidly expanding in inflammatory contexts.^45^ This pathway also appears important in humans, as administering an anti‐*β*_8_ blocking antibody following co‐transfer of human peripheral blood mononuclear cells and autologous Treg cells to immune‐deficient mice abrogated Treg‐mediated immunosuppression and exacerbated graft‐versus‐host disease.^46^

### TGF‐*β*‐dependent intestinal tissue‐resident T cells

TGF‐*β* has also been implicated as a critical factor involved in the establishment and maintenance of intestinal tissue‐resident memory (Trm) cells, a non‐circulating memory T‐cell population that reside in peripheral tissues.^47^, ^48^ A seminal study by Zhang and Bevan demonstrated that TGF‐*β* was essential for the formation and maintenance of intestinal Trm via a two‐step process by firstly inhibiting the *α*_4_ *β*_7_‐mediated recruitment of splenic T cells into the gut and subsequently inducing the expression of tissue residency markers, such as CD103 and CD69 on T cells situated in the intestine.^49^ Of note however, a *bona fide* population of CD103^–^ intestinal Trm cells has also been described, whose differentiation occurs independently of TGF‐*β*.^50^ More recently, expression of the TGF‐*β*‐activating integrin *α*_v_ *β*_6_ by intestinal epithelial cells has also been shown to be critical for the maintenance of CD8^+^ Trm cells. In their study, Mohammed *et al.* 51 demonstrated that although the infiltration of effector cells into the epithelial layer during acute infection was comparable between wild‐type and *Itgb6^–/–^* mice, following contraction of this initial response, the numbers of antigen‐specific cells that were retained within the intestinal epithelium, but not the lamina propria, were significantly reduced in the absence of *α*_v_ *β*_6_‐mediated TGF‐*β* activation.

**Figure 2:**
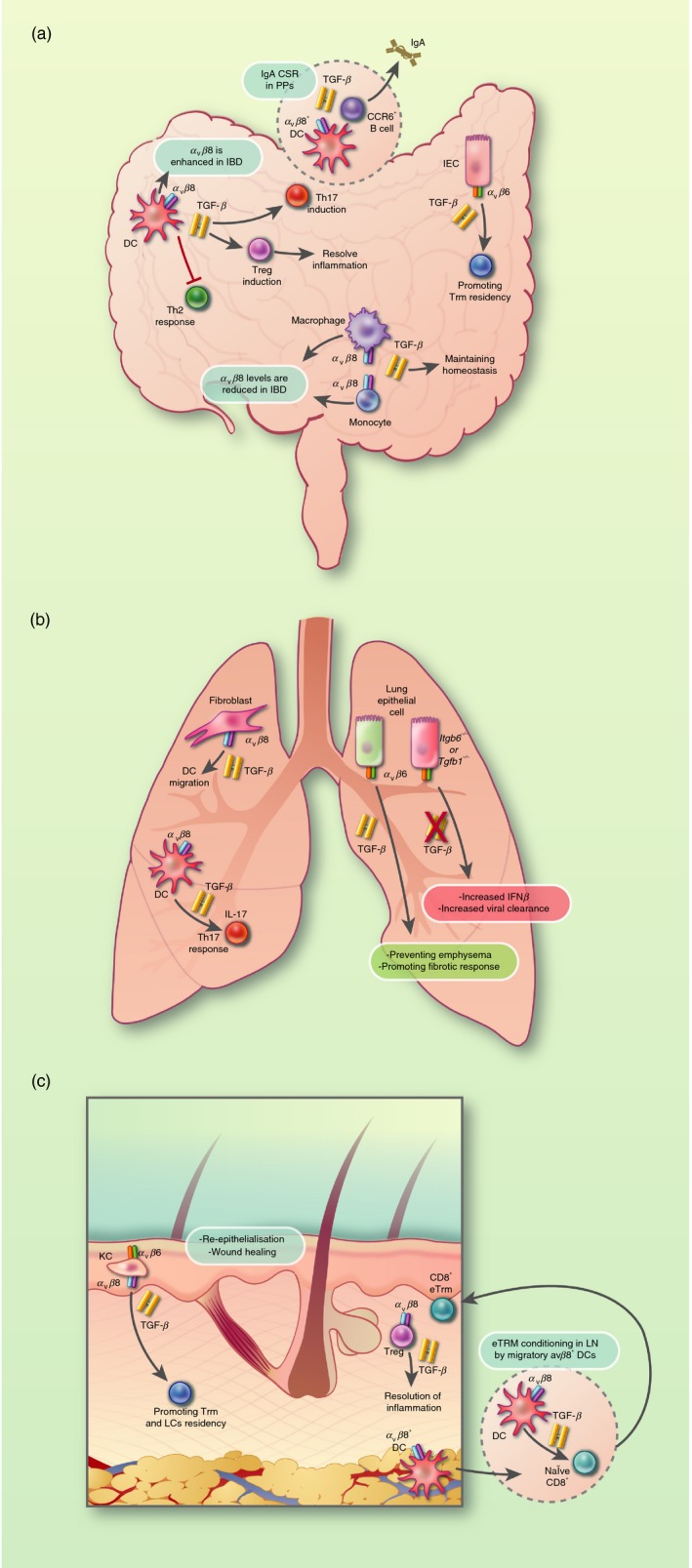
Transforming growth factor‐*β* (TGF‐*β*) activated by integrins *α*_v_ *β*_6_ and *α*_v_ *β*_8_ has important functions at barrier sites. (a) Dendritic cells (DCs) are capable of activating latent TGF‐*β* through their expression of the integrin *α*_v_ *β*_8_, which modulates intestinal CD4^+^ T helper (Th) cell responses and Foxp3^+^ regulatory T (Treg) cells. In inflammatory bowel disease (IBD), *α*_v_ *β*_8_ expression is enhanced on DCs, whereas it is reduced on monocytes and macrophages. TGF‐*β* activation by integrin *α*_v_ *β*_6_ by intestinal epithelial cells (IECs) has been shown as a critical factor involved in the maintenance of intestinal tissue‐resident memory (Trm) cells. (b) In the lung, activation of TGF‐*β* by *α*_v_ *β*_8_‐expressing fibroblasts has been shown to drive DC chemotaxis. Moreover, integrin *α*_v_ *β*_8_‐expressing DCs appears to be required for the differentiation of Th17 cells. Integrin *α*_v_ *β*_6_‐mediated activation of latent TGF‐*β* by lung epithelial cells is a critical component of pulmonary homeostasis via preventing emphysema, whereas excessive TGF‐*β* signalling can facilitate pulmonary fibrosis in idiopathic pulmonary fibrosis (IPF) and promote infection. (c) Integrins *α*_v_ *β*_6_ and *α*_v_ *β*_8_ play an essential role in maintaining the cutaneous barrier in the skin. *α*_v_ *β*_6_‐mediated activation of latent TGF‐*β* by keratinocytes (KCs) is essential for re‐epithelialization and wound healing. Moreover, activation of latent TGF‐*β* by integrins *α*_v_ *β*_6_ and *α*_v_ *β*_8_ by KC in the epidermis are important for the maintenance of both Trm and Langerhans cells (LCs) in the skin. *α*_v_ *β*_8_ expression on regulatory T (Treg) cells is required to prevent overt effector T‐cell responses during acute inflammation. Migration of integrin *α*_v_ *β*_8_‐expressing DCs to skin‐draining lymph nodes is also important in imprinting CD8^+^ T cells to become CD8^+^ Trm cells.

Hence, regulation of TGF‐*β* activation by integrins plays a crucial role in controlling a number of facets of TGF‐*β* function in the intestine (Fig. 2a), with several of these pathways appearing dysregulated during IBD. Hence, a better understanding of how TGF‐*β* activity is controlled in the gut in a context‐specific manner may identify potential targets for modulating TGF‐*β* function in IBD.

## TGF‐*β* activation in the lung

### TGF*β* and its activation as a mediator of tolerance to inhalable allergens

As with other barrier sites, the pulmonary mucosa is constantly exposed to a plethora of harmless environmental antigens, which are inhaled on a daily basis and to which the immune system must remain in a state of immunological hyporesponsiveness to prevent unwarranted effector responses and maintain normal tissue function. Perturbations in the mechanisms that maintain this steady‐state tolerance to innocuous antigens, such as pollen, pet dander and dust mite excretions, can result in the development of airway hyper‐reactivity, characterized as the onset of overt inflammatory responses culminating in airway obstruction. As is the case with oral tolerance to ingested antigens in the gut, Treg cells and TGF‐*β* are fundamental in the prevention of exacerbated type 2 responses in the airways, which are characteristic of allergic inflammation and abrogation of TGF‐*β* signalling can re‐capitulate inflammatory phenotypes in a number of experimental models.^52^, ^53^, ^54^, ^55^, ^56^, ^57^ Lung‐resident macrophages, like tolerogenic intestinal DCs, have been shown to co‐express TGF‐*β* and RALDH constitutively and are capable of inducing the differentiation of Foxp3^+^ T cells from naive precursors in *in vitro* culture systems.^58^ Moreover, several studies have demonstrated that depletion of these macrophages *in vivo* enhances allergen‐induced responses, with Zasłona *et al.* reporting a concomitant decrease in TGF‐*β* concentrations in bronchoalveolar fluid.^59^, ^60^, ^61^

Interestingly, TGF‐*β*‐activating integrins also play a central role in maintaining the balance between effector and tolerogenic responses in the lung, as demonstrated by work showing that loss of *α*_v_ *β*_6_ resulted in enhanced metalloproteinase activity and degradation of extracellular matrix components, culminating in the development of murine emphysema.^62^

### TGF‐*β* activation as a driver of disease in the lung

Despite its ordinarily tissue‐protective roles, excessive TGF‐*β* signalling in the lung can have detrimental consequences and can facilitate pulmonary fibrosis owing to its ability to stimulate collagen deposition and the generation of new extracellular matrix.^63^ In the case of idiopathic pulmonary fibrosis (IPF) this seems to occur in an integrin *α*_v_ *β*_6_‐dependent manner, as blockade or genetic ablation of *α*_v_ *β*_6_ in mice attenuated fibrotic sequelae in a variety of pre‐clinical models.^14^, ^64^, ^65^ This process appears to involve a positive feedback loop in which *α*_v_ *β*_6_ expression is first induced by TGF‐*β*, which is found at elevated concentrations in the lungs of IPF patients.^66^ Similarly, enhanced airway TGF‐*β* levels are a feature of both asthma and chronic obstructive pulmonary disease (COPD), where it is associated with airway remodelling characteristic of disease pathogenesis.^67^

Integrin‐mediated TGF‐*β* activation has also been indicated in the pathogenesis of asthma. In a pre‐clinical model of airway hyper‐responsiveness in which mice exhibit asthma‐like symptoms, Kudo *et al.* demonstrated that activation of latent TGF‐*β* by integrin *α*_v_ *β*_8_‐expressing DCs was required for the differentiation of Th17 cells following sensitization with inhaled antigens. Subsequent intra‐nasal challenge resulted in interleukin‐17A‐dependent smooth muscle contraction and airway constriction, a response that did not occur in mice lacking integrin *α*_v_ *β*_8_ on their DCs.^68^ Interestingly, Th2 cell numbers were not altered in mice lacking integrin *α*_v_ *β*_8_‐mediated TGF‐*β* activation by DCs.^68^ Given that TGF‐*β* is a known inhibitor of Th2 cell formation, these results show that TGF‐*β* activation by integrins allows context‐specific TGF‐*β* function; in this instance promoting Th17 function without affecting Th2 cells. Additionally, expression of another integrin, *α*_v_ *β*_5_, by airway smooth muscle cells has been proposed to be important in activating TGF‐*β* and promoting pathology in murine models of asthma.^69^ Hence, limiting the concentrations of bioactive TGF‐*β* by selectively targeting the integrins involved in its activation may represent a promising strategy by which to attenuate pulmonary fibrosis and asthma without compromising tolerance to innocuous antigens.

### TGF‐*β* production and activation by pulmonary epithelial cells can limit effector responses during lung infection

In addition to these deleterious roles in fibrosis and asthma, recent work has suggested that TGF‐*β* serves to promote viral infection, and can be used to evade type I interferon‐mediated anti‐viral immune responses. Again, integrins appear to play a key role in facilitating such responses and Meliopoulos *et al.* 70 demonstrated enhanced protection against influenza virus, Sendai virus and *Streptococcus pneumoniae* in mice lacking integrin *α*_v_ *β*_6_, a phenotype that was attributed to enhanced activation status and type I IFN signalling in alveolar macrophages and could be reversed upon exogenous administration of TGF‐*β*. Similarly, Denney *et al.* 71 recently reported that conditional deletion of *Tgfb1* in bronchial epithelial cells led to reduced viral burdens and infection‐associated pathology following infection with influenza virus owing to elevated interferon‐*β* responses. Interestingly, the same group previously demonstrated that epithelial‐derived TGF‐*β* actually exacerbated inflammatory sequelae by augmenting numbers of allergen‐induced pulmonary innate lymphoid cell type 2, further highlighting the dichotomous roles of TGF‐*β* within the lung microenvironment.^72^ Aside from exhibiting both pro‐inflammatory and anti‐inflammatory functions, the finding by Meliopoulos *et al.* 70 that the lung microenvironment of *α*_v_ *β*_6_‐deficient animals imprinted an altered developmental phenotype on alveolar macrophages, which could be reversed upon administration of exogenous TGF‐*β*, suggest that the activation of latent TGF‐*β* by integrin *α*_v_ *β*_6_ may in fact play a key role in the ontogeny of these cells.

### Targeting TGF‐*β* activity in lung disease

As a result of its pro‐fibrotic properties, together with its potent immune suppressive functions, TGF‐*β* has emerged as a promising therapeutic target for the treatment of fibrosis and cancer. Pirfenidone is a small‐molecular‐weight phenyl‐substituted pyridinone with anti‐inflammatory and anti‐fibrotic effects, which include decreased fibroblast proliferation and TGF‐*β* signalling. It has been approved for clinical use for the treatment of IPF and has been shown to attenuate TGF‐*β* function and improve vital capacity in IPF patients.^73^ However, because of the pleiotropy of TGF‐*β* in maintaining normal tissue function and prevention of deleterious responses to self‐antigens or innocuous stimuli, there is a concern that systemic blockade of TGF‐*β* signalling might have severe complications and result in dysregulated immune responses. Therefore, an alternative approach that might attenuate, rather than completely ablate, TGF‐*β* responsiveness is highly desirable. Indeed several approaches targeting specific components of the TGF‐*β* signalling pathway are currently under investigation. One such strategy might involve targeting the activation of TGF‐*β* by some of the aforementioned integrins, thereby limiting the amount of active TGF‐*β* available to target cells. BG0001, a humanized monoclonal antibody targeting the activation of TGF‐*β* by integrin *α*_v_ *β*_6_ has recently demonstrated a decrease in active TGF‐*β* signalling in a phase 2A trial and has subsequently undergone phase 2B trials for IPF (clinical trial # NCT01371305).^74^

Similarly, targeting *α*_v_ *β*_8_ may be efficacious for the treatment of certain pulmonary indications. Activation of TGF‐*β* by *α*_v_ *β*_8_‐expressing fibroblasts has been shown to drive DC chemotaxis in response to exogenous stimuli associated with COPD.^75^, ^76^ Recently, the potential efficacy of an integrin *α*_v_ *β*_8_‐targeted therapy for pulmonary fibrosis has been demonstrated in transgenic mice expressing human *α*_v_ *β*_8_. In their study, Minagawa *et al.* 77 successfully attenuated fibrotic inflammation in response to a number of stimuli including allergens, tobacco smoke and pro‐inflammatory cytokines by administering a humanized monoclonal antibody specific for *α*_v_ *β*_8_. Taken together, these studies highlight the promising potential of integrin‐targeted therapies for the treatment of various lung pathologies.

### Role of TGF‐*β* in the establishment of long‐lived lung‐resident memory T cells

Similar to the gut, another function of pulmonary TGF‐*β* signalling is its role in regulating the expression of tissue residency markers on T cells, thereby enabling them to differentiate into long‐lived Trm cells. The establishment of these cells in a TGF‐*β*‐dependent manner is one of the primary objectives of mucosal vaccination and has been shown to be essential for hetero‐subtypic protection against influenza challenge.^78^ In humans, the imprinting of a Trm signature, including up‐regulation of the tissue residency marker CD103, on pulmonary T cells is a specialized function attributed to CD1c^+^, as opposed to CD141^+^, DCs and is dependent on TGF‐*β*.^79^ As mentioned previously, this CD1c^+^ DC subset has been shown to activate TGF‐*β* via expression of integrin *α*_v_ *β*_8_ in the gut, a function that is essential for the prevention of intestinal immune dysfunction.^40^ Taken together, these findings suggest that integrin *α*_v_ *β*_8_‐mediated activation of latent TGF‐*β* by CD1c^+^ DCs may also regulate the formation of protective Trm cells at the pulmonary mucosa.

Overall, data generated to date show that TGF‐*β*, and the control of its activity by integrins, is vital in the regulation of responses in the lung in both health and disease (Fig. 2b). Hence, blockade of TGF‐*β* activation by integrins is an attractive therapeutic target in pulmonary fibrosis and COPD, as well as during pulmonary infections, with the potential to block context‐specific TGF‐*β* function in disease without compromising the tissue‐protective effects of the cytokine.

## TGF‐*β* activation in the skin

TGF‐*β*, and several components involved in its signalling pathway, play an essential role in regulating the cutaneous barrier. Such pathways are generally up‐regulated in response to injury or inflammatory stimuli to help promote wound healing or attenuate inflammatory responses by inducing cell cycle arrest or the apoptosis of effector cells.^80^ Additionally, TGF‐*β* is critical in the formation of Langerhan's cells, and in preventing their migration out of the epidermis.^81^ As in gut and lung, integrin‐mediated TGF‐*β* activation is critical in controlling the function of TGF‐*β* in the skin. For example, although integrin *α*_v_ *β*_6_ and *α*_v_ *β*_8_‐mediated TGF‐*β* activation by keratinocytes are not required to prevent spontaneous skin inflammation,^82^ *α*_v_ *β*_6_‐mediated activation of latent TGF‐*β* by keratinocytes has been shown to be essential for re‐epithelialization and optimal wound healing both *in vitro* and *in vivo*.^83^ Moreover, Treg expression of *α*_v_ *β*_8_ is required to prevent overt effector T‐cell responses during acute inflammation, as mice lacking expression of integrin *α*_v_ *β*_8_ on Foxp3^+^ Treg cells could not restrain effector CD4^+^ and CD8^+^ cellular responses in mouse models of delayed hypersensitivity, leading to exacerbated inflammation and thickening of the cutaneous barrier.^45^ Additionally, integrins *α*_v_ *β*_3_ and *α*_v_ *β*_5_ are up‐regulated in dermal fibroblasts from scleroderma patients and are proposed to promote myofibroblast formation through activation of TGF‐*β*.^15^, ^16^

### Activation of latent TGF‐*β* determines skin residency

As with other barrier sites, TGF‐*β*, in synergy with transcription factors and other cytokines such as interleukin‐15, is a key factor involved in retaining memory T cells in the skin as long‐lived Trm cells.^84^, ^85^ Co‐ordinated activation of latent TGF‐*β* by integrins *α*_v_ *β*_6_ and *α*_v_ *β*_8_, which are expressed in a reciprocal fashion on keratinocytes found at distinct anatomical locations in the epidermis has been implicated in the maintenance of both Trm and Langerhans cells in the skin of both mice and humans.^51^ Recently, the expression of α_v_ integrins, specifically *α*_v_ *β*_8_, by migratory DCs in the skin has been shown to be essential for the formation of CD103^+^ skin‐resident CD8^+^ T cells. In their study, Mani *et al.* demonstrated that mice lacking expression of all *α*_v_ integrins, or just *α*_v_ *β*_8_ alone, on DCs exhibited significantly reduced numbers of CD103^+^ epidermal Trm cells both at steady‐state and following inflammation or immunization. Interestingly, these *α*_v_ integrin‐expressing DCs were shown to first migrate to the lymph node, where their ability to activate latent TGF‐*β* pre‐conditioned naive CD8^+^ T cells for subsequent tissue residency.^86^ In addition to this well‐established role for integrin‐activated TGF‐*β* in the context of tissue residency, a recent study by Dan Kaplan and colleagues has shown that local activation of latent TGF‐*β* in the skin also regulates the pool of recirculating memory CD8^+^ T cells following primary infection and is required for their persistence as peripheral or central memory T cells. In their study, complete ablation of integrins *α*_v_ *β*_6_ and *α*_v_ *β*_8_ on keratinocytes led to impaired TGF‐*β* activation, resulting in decreased numbers of peripheral and central memory T cells, which significantly compromised protection from a secondary challenge with Vaccinia virus.^87^

In summary, integrin‐mediated TGF‐*β* activation is key in regulating various aspects of skin biology (Fig. 2c), with further work required to determine how targeting these pathways may be useful during wound healing and infection.

## Conclusion and perspectives

In this review, we have discussed pleiotropic effects of TGF‐*β* and the source of activation of TGF‐*β* that can differ in certain contexts at barrier sites. These pathways highlight the multi‐faceted manner in which barrier site homeostasis is maintained and orchestrated by several highly specialized cell types. Given the importance of TGF‐*β* in many different tissues and biological contexts, this cytokine has emerged as a promising therapeutic target in disorders such as fibrosis and cancer. However, given the broad importance of TGF‐*β* in health, global antagonism of TGF‐*β* signalling may have detrimental consequences, which pose a major challenge for therapeutic targeting. Therefore, TGF‐*β*‐activating integrins, specifically *α*_v_ *β*_6_ and *α*_v_ *β*_8_, become important potential targets to affect TGF‐*β* function in a more context‐specific manner. However, more work is needed to understand the relative contributions of these integrins on specific cell types, any non‐TGF‐*β*‐related functions they play that will also be blocked when targeted, and how their expression and function are regulated during health and disease. This will be an important step towards the translation of ‘promising’ targets into clinically effective therapeutics for disease.

## Disclosures

The authors declare there are no competing interests.
